# *Caenorhabditis elegans* as a Useful Model for Studying Aging Mutations

**DOI:** 10.3389/fendo.2020.554994

**Published:** 2020-10-05

**Authors:** Siwen Zhang, Fei Li, Tong Zhou, Guixia Wang, Zhuo Li

**Affiliations:** Department of Endocrinology and Metabolism, First Affiliated Hospital of Jilin University, Changchun, China

**Keywords:** IGF-1, AMPK, mTOR, dietary restriction, drug screening

## Abstract

The *Caenorhabditis elegans* genome possesses homologs of about two-thirds of all human disease genes. Based on its physiological aging characteristics and superiority, the use of *C. elegans* as a model system for studies on aging, age-related diseases, mechanisms of longevity, and drug screening has been widely acknowledged in recent decades. Lifespan increasing mutations in *C. elegans* were found to delay aging by impinging several signaling pathways and related epigenetic modifications, including the insulin/IGF-1 signaling (IIS), AMP-activated protein kinase (AMPK), and mechanistic target of rapamycin (mTOR) pathways. Interestingly, dietary restriction (DR) has been shown to increase the lifespan of numerous metazoans and protect them from multiple age-related pathologies. However, the underlying molecular mechanisms are unclear. In recent decades, *C. elegans* has been used as a unique model system for high-throughput drug screening. Here, we review *C. elegans* mutants exhibiting increased in lifespan and age-dependent changes under DR, as well as the utility of *C. elegans* for drug screening. Thus, we provide evidence for the use of this model organism in research on the prevention of aging.

## Introduction

The eukaryotic multicellular organism *Caenorhabditis elegans* which has completely sequenced genetic profile, is an established genetic model organism (1), that can be used to study aging. The use of *C. elegans* as a model system to recapitulate most human diseases in recent decades is invaluable for experimental research at both the metabolic and genomic levels *in vivo* (2, 3). In addition, research studies on aging using *C. elegans* have provided desirable outcomes in identifying molecular signals, transcriptional regulators, and epigenetic modifications associated with longevity broadening our ability to understand how organisms age. In this review, we aim to provide an overview of the established and current novel concepts on transcriptional and epigenetic regulators in the field of research on aging using the model organism *C. elegans* and elucidate how dietary restrictions influence these specific regulators, as well as discuss the application of *C. elegans* in drug screening studies.

## Background, Advantages, and Limitations of *C. elegans* for Studies on Aging

### Basic Features and Age-Dependent Changes of *C. elegans*

*C. elegans* is a free-living, harmless nematode that feeds on microorganisms. It is particularly economical and easy to maintain in laboratory settings. Adult *C. elegans* are 1 mm long self-fertilizing hermaphrodites with a 2.5–4 days reproductive cycle at room temperature, and a mean lifespan of approximately 18–20 days when cultured at 20°C (4–7). After hatching, *C. elegans* can either develop directly to four larval stages (L1–L4) or proceed with the dauer larval stage after the L2 larval stage, instead of the L3 larval stage. The dauer larval stage is a developmentally arrested dispersal stage used to survive adverse conditions. Once the adverse conditions subside, *C. elegans* can recover and molt into the L4 larval stage, continuing normal development (8). In worms, features associated with aging could result in less active, uncoordinated movements, torpor, cessation of reproduction, and accumulation of auto-fluorescent deposits in cells (7, 9, 10)^1^. Clear age-dependent humanlike physiological changes at the tissue, cellular, and molecular levels make *C. elegans* a valuable model for research in the field of aging.

Aged *C. elegans* display a decline in their anatomical and functional features, including tissue integrity, motility, learning and memory, and immunity. The primary age-dependent changes at the tissue level include changes in the reproductive, nervous, and muscular systems. The rate of reproduction marked decreases and the structure of the reproductive system deteriorates with age. Oocyte size and quality also deteriorate with advancing age (11). *C. elegans* displays structural changes and functional deterioration of neurons during aging. Blebbing and branching structures can be seen in aged touch receptor neurons, indicating that the synaptic integrity degenerates with aging (12, 13). In addition, it has been widely reported that the loss of muscular integrity, sarcomeres density, and regular orientations in aged *C. elegans* result in impaired motility and an abnormal appearance (14).

At the cellular level, the primary age-dependent changes in *C. elegans* generally include diminishing integrity of nuclei and increased relative size of the nucleoli; however, these changes may vary depending on the tissue types. Moreover, mitochondria undergo age-dependent structural and functional changes, including mitochondrial fusion and increased mitochondrial fragmentation, which is consistent with changes in mitochondrial DNA copy numbers and oxygen consumption rates (15). The capacity of the unfolded protein response of endoplasmic reticulum (ER^UPR^) seems to be reduced in aged *C. elegans*. The ER^UPR^ is activated and tasked with degrading the misfolded proteins under various stress conditions (16). Reduced ER^UPR^ process result in misfolded proteins increasing and leading to age-related diseases.

The *C. elegans* genome possesses homologs of about two-thirds of all human disease genes. In a previous study, Zhao et al. performed functional analysis of 143 essential genes and found that 108 of them were human orthologs. Of these, 97 genes were associated with 1,218 different diseases (17). Age-associated molecular changes provide more information and serve as valuable biomarkers for aging. Many changes in aging-associated gene expression, which increase lifespan but decrease with age, have been identified during *C. elegans* aging (18). The quality of RNA control mechanisms, such as non-sense-mediated mRNA decay (NMD) activity in various organs and tissues, decline with advancing age in *C. elegans*. Also, the increased levels of introns and unannotated regions in the mRNAs denote a decrease of mRNA splicing fidelity in aged worms (19). Protein homeostasis associated with age-related diseases declines during aging (20). It has been reported that proteins involved in nucleosome assembly, ER nuclear signaling, and the response to unfolded proteins increase, whereas the abundance of proteins involved in metabolism (fatty acid, carbohydrate, and amino acid) decreases during aging (21). In addition, the levels of amino acid metabolites also change with age (22). The effects resulting from gene expression changes during aging are not yet fully understood. Further studies will be needed to comprehensively elucidate the roles of age-dependent changes in the levels of amino acid in aging and longevity.

### Advantages of *C. elegans* on Aging Study

*C. elegans* is an excellent model organism used for aging research. The ease of its maintenance in the laboratory, transparent body for anatomical observation, high genetic homology (60–80%) with humans, availability of complete genome sequence, conserved biological molecular responses, high fertility rates (~250 eggs/worm within several days), and the availability of molecular biology tools (i.e., transgenic, gene knockouts, and RNAi knockdowns) make *C. elegans* a useful model for the study of aging mutations (23). In addition, the short lifespan of this organism (~3 weeks) and small size are favorable for the screening of anti-aging drugs due to the reduced experimental costs and their usability for a high throughput screening experiments (24). Moreover, experiments with *C. elegans* are free of ethical concerns. Many breakthrough discoveries in the field of aging research have been achieved using *C. elegans* because of these advantages (25).

### Limitations of *C. elegans* on Aging Study

*C. elegans* shows many desirable features for aging studies, however, it still has some limitations as a model organism compared to other mammals. Firstly, *C. elegans* lack certain anatomical features of mammals, including a blood transport system, a blood-brain barrier, a first-pass metabolism process in the liver, and blood filtration in the kidney, which may be specific for certain signal pathways or epigenetic effects (26). As a model system to predict human research outcomes, the lack of DNA methylation, an epigenetic tag that possibly has a greater function in mammals than in nematodes, is another limitation of *C. elegans* (27). In addition, the lack of long-range transcriptional regulation makes it inadaptable for studying the relevant mechanism in other animal species; however, it is recommended as a simplified model for studies on signal mapping mechanisms (27).

## Transcriptional Regulators and Epigenetic Regulation in *C. elegans* Aging

Previous studies have identified several loci that increase the lifespan of *C. elegans* when mutated. The molecular genetics of this organism is well-established and has been strongly supported by a fully sequenced genome, which provides insight regarding the entire 959 somatic cells that constitute it. Thus, far, over 50 genes that control aging in *C. elegans* have been identified (50). Of these, many have homologs in other organisms. Different upstream signals stimulate partially overlapping sets of downstream mediators and processes that ultimately extended the lifespan. Meanwhile, epigenetic regulation in cooperation with transcriptional regulators influence the functions of cells and the fate of organisms, and could act as markers of aging (51). Epigenetic regulation involves DNA methylation, chromatin remodeling, post-translational modifications (PTMs) of histones, and non-coding RNA transcription (28). Here we discuss longevity mechanisms related to transcriptional regulators of metabolic networks and epigenetic regulation in *C. elegans*.

### The Insulin/IGF-1 Signaling Pathway

**Figure 1:**
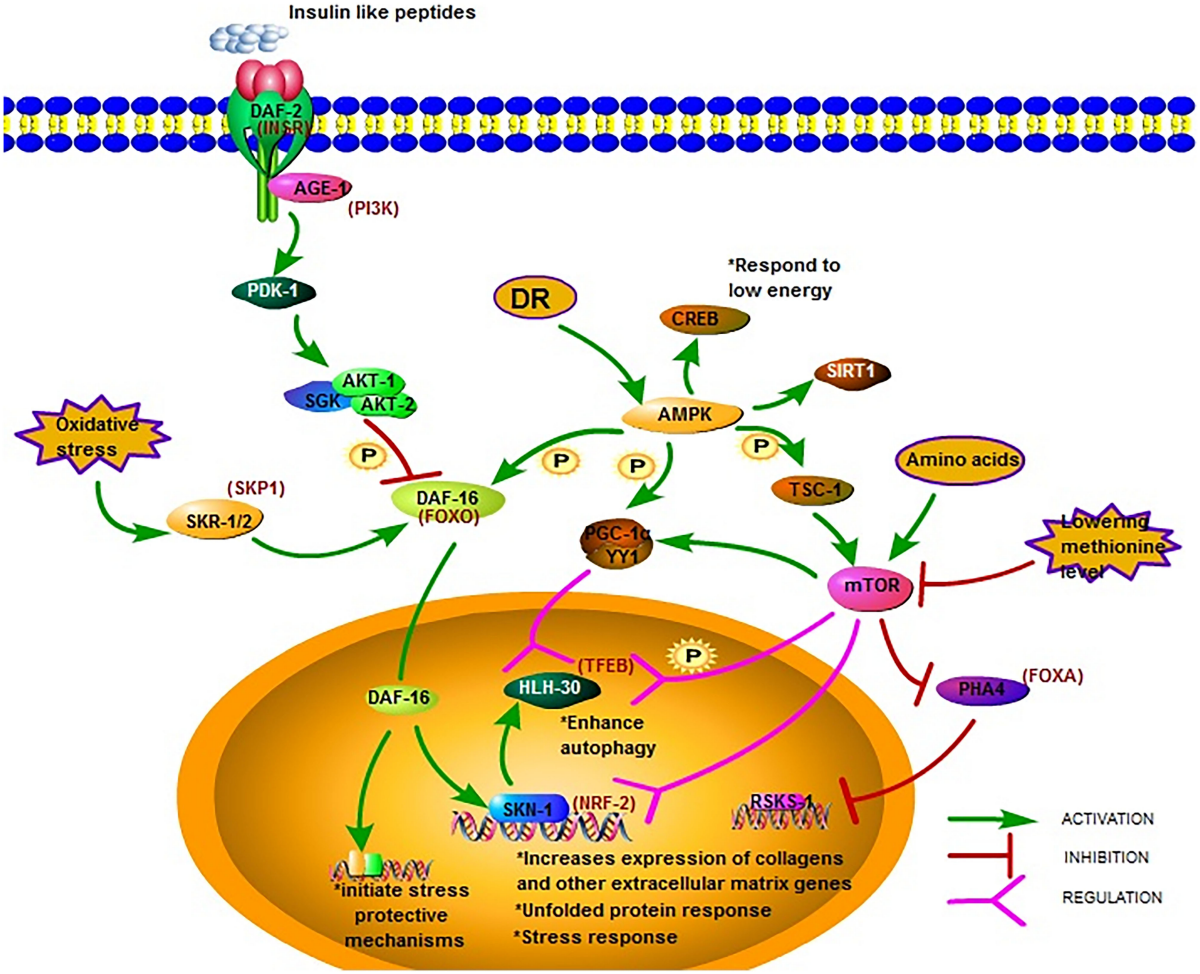
Major longevity pathways and longevity-associated transcription factors in *C. elegans*. The IIS pathway is one of the most studied longevity pathways. DAF-2, AGE-1, and DAF-16 are its three key genes. Mutations in DAF-2 and AGE-1 and low IIS activity prolong lifespan *via* DAF-16 and downstream gene expression. AMPK is another crucial metabolic energy sensor that links nutrient availability to lifespan by binding and phosphorylating a set of transcriptional (co)activators. The mTOR pathway is another critical pathway that links nutrient availability and metabolism to longevity; however, its mechanism remains to be fully elucidated (18, 28–49).

The insulin/IGF-1 signaling (IIS) pathway is one of the most studied longevity pathways (52, 53). This pathway has three key genes, namely *daf-2, age-1*, and *daf-16*. While *daf-2* encodes a homolog of the mammalian insulin/IGF-I receptor (INSR) (54), *age-1* encodes a homolog of the catalytic p110 subunit of mammalian phosphoinositide 3 kinase (PI3K) (55, 56). In *C. elegans, daf-16* is widely expressed and encodes a homolog of human forkhead box O (FOXO) transcription factor (54). The greatest increases in lifespan due to mutations in single genes have been reported for *daf-2* and *age-1* (57–59). The mean survival of long-lived *daf-2* and *age-1* mutants was around 15% longer than that of the wild-type (60). Mutations in *daf-2* and *age-1* resulted in arrested larvae and forced larvae into the dauer stage, increasing the lifespan of the nematodes, as well as enhancing stress resistance (61). The prolonged lifespan of *daf-2* and *age-1* mutants were dependent on DAF-16(FOXO) (Figure 1). In worms with the *daf-2* mutation, the activity of the IIS pathway decrease leading to the phosphorylation of DAF-16 by AKT-1/2 and the translocation of DAF-16 into the nucleus to bind and initiate expression of target genes, Consequently the lifespan of the worm is prolonged and stress-protective mechanisms, including the unfolded protein response and oxidative stress responses, are initiated (29). The transcription factor DAF-16(FOXO) induces juvenile *C. elegans* to develop into dauer larvae, which represents diapause that allows this organism to withstand harsh conditions. Reactive oxygen species (ROS) can modulate the import of DAF-16 into the nucleus *via* disulfide bond formation with transportin-1 (IMB-2) (62). The main role of DAF-2(AGE-1) signaling is to antagonize *daf-16* (63, 64). It has been reported that the metabolic, longevity, and developmental defects caused by *daf-2* and *age-1* mutations are antagonized by *daf-16* mutations (64–66). DAF-16 may directly regulate the transcription of the genes necessary for the increased longevity observed in *age-1* and *daf-2* mutants (29). As such, one research direction is to identify genes under the control of DAF-16. The strong association between FOXO expression and lifespan has been reported by several studies on humans and is considered a promising therapeutic target to promote longevity.

Reduced IIS promotes *C. elegans* longevity through the NF-E2-related factor (NRF2) ortholog SKN-1, which operates as a genetically distinct program from the dauer pathway and in parallel to DAF-16 (30). The transcription factor SKN-1(NRF2) is considered an important regulator of detoxification and oxidative stress responses in *C. elegans*; SKN-1 (NRF2) prominently increases the expression of collagens and other extracellular matrix genes when IIS level is decreased (18). The expression of the *skn-1* target gene upon oxidative stress can also be promoted by SKR-1/2, which is the ortholog of the mammalian SCF-ubiquitin ligase complex member SKP1. Furthermore, it has been reported that DAF-16 can regulate SKN-1 transcription and that *daf-16* is a target of SKR-1/2, indicating that SKN-1 mediated stress resistance may not be necessary associated with longevity (31) (Figure 1).

Epigenetic regulation on specific targets of metabolic signaling pathways can alter the lifespan. The demethylase UTX-1, which targets genes such as *daf-2* on the IIS pathway, regulates the lifespan in *C. elegans*. It reduces DAF-16(FOXO) levels, as mentioned above, compromising cellular maintenance processes and weakening the ability to resist stress in *C. elegans*, thus inducing an aging-related decline in cellular functions (32). Besides, LncRNAs with a variable length spanning from 200 base pairs up to several kilobases are important for cell function, because it can target classic signal pathways such as IIS. For example, the LncRNA tts-1 extends lifespan by reducing ribosome levels in the *daf-2* mutant *C. elegans* (28). IIS conserved in both insects and mammals; the genetic and biological characteristics of the IIS pathway in aging were successfully translated to mammals and humans (67).

### AMP-Activated Protein Kinase Signaling Pathway

Different signal pathways could regulate each other by PTMs. For example, the post-translational modifications of DAF-16 include phosphorylation by AAK-2 (AMPK), which belongs to another important signal pathway related to metabolic energy. AMP-activated protein kinase (AMPK) is a crucial metabolic energy sensor linking nutrient availability with lifespan (68). The AMPK encoding gene is *aak-2* in *C. elegans*. As a master regulator of cellular energy homeostasis, AMPK is required for the metabolic adjustment during the starved, developmentally quiescent diapause phase of *C. elegans* (69). The overexpression of AMPK extends lifespan, as shown in *C. elegans* (33). Upon activation, AMPK binds and phosphorylates a set of transcriptional coactivators, including PGC-1α, FOXO, and SIRT1, and the actions of AMPK activation at least partially overlap with sirtuin activation (34). Similar to the mechanism in the mammalian system, *aak-2*-mediated longevity requires the downregulation of the IIS pathway and the subsequent upregulation and translocation of DAF-16(FOXO). Post-translational modifications of DAF-16 include its phosphorylation by AMPK (70). AMPK modulation of lifespan has been shown to occur also *via* CREB-regulated transcriptional coactivators in response to low levels of energy (35) (Figure 1).

Starvation can induce long-term consequences through epigenetic change. AMPK is required for metabolic adjustment by blocking specific chromatin modifications and epigenetic changes in *C. elegans* larvae to resist starvation (69). The recovery of *C. elegans* after starvation in the early larval stage would be impaired with the absence of AMPK, and could become sterile. AMPK might affects heritable aspect including germline gene expression or genomic integrity, which need further research.

Recently, another of transgenerational lifespan regulation paradigm was shown in *C. elegans*. Lacking DNA methylation to activate histone modifications such as reduced methylation of Lys4 of histone H3 (H3K4me) are characters of actively transcribed genes (71). DNA methylation usually occurs at 5-methyl cytosine (5 mC) and result in transcriptional repression (72). In *C. elegans*, 5 mC methylation is rare, while methylation on N6 adenine (6 mA) is prevalent of silence DNA repeats (73). NMAD-1 (MT-A70 family) and DMAD (DNA 6 mA demethylase, TET ortholog) are 6 mA demethylases and can regulate 6 mA levels in *C. elegans* (74). DAMT-1 (AlKB family), a likely 6 mA methyltransferase, can also control the epigenetic inheritance of phenotypes which associated with the loss of the H3K4me2 demethylase SPR-5 (CoREST/LSD1 ortholog) (75). Deletion of the SPR-5, 6 mA increases across generations and can lead to a progressive transgenerational loss of fertility, the worms become sterile after several generations (76). Longevity can also be transmitted across generations by this kind non-genetic factors. Greer et al. demonstrated that deletion of the *spr-5* in *C. elegans* causes a trans-generational increase in lifespan through mis-regulation and activation of lifespan-regulating signaling pathway (77). It is reported that SPR-5 has numerous consensus AMPK phosphorylation sites (69), but whether SPR-5 is yet another functional nuclear target of AMPK remains to be established.

### Mechanistic Target of Rapamycin Signaling

Another critical pathway linking nutrient availability and metabolism to longevity is the mechanistic target of rapamycin (mTOR) pathway. This pathway is activated upon the increase of intracellular amino acids or during growth factor stimulation and modulates a set of downstream signaling pathways that manage cell proliferation, cell growth, motility, survival, and protein synthesis (78). Studies in *C. elegans* have shown that the inhibition of mTOR activity prolongs the worm lifespan (79). Longevity mediated by inhibiting the mTOR pathway is most likely distinct from the IIS pathway, and an overlapping mechanism may also occur between these two pathways.

mTOR regulates mitochondrial gene expression and control energy- and nutrient related mitochondrial respiration by activating the peroxisome proliferator-activated receptor coactivator-1 α (PGC-1 α) forming a complex with the transcription factor Yin-Yang 1 (YY1) to promote the expression of related genes (36) (Figure 1). One possible downstream pathway that serves as a shared longevity mechanism between the IIS and mTOR pathways is autophagy (31). Recent studies show that the effect of defective mitochondria on cells can be prevented by mTOR during aging *via* the mitophagy process, which is a kind of autophagy, which targets the mitochondria. Beside PGC-1 α signaling and mitophagy, mTOR may also influence mitochondria *via* SKN-1 signaling (37).

The transcription factor EB (TFEB), found in *C. elegans* as HLH-30, is an autophagy enhancer that regulate gene expression related to autophagy and lysosomal (38). In the nucleus, the localization of HLH-30(TFEB) is modulated *via* phosphorylation by mTOR and the function is regulated *via* competition with MXL-3/MAX and by its interaction with the Mondo-complex (Figure 1). Potential nuclear interactions between HLH-30 (TFEB) and DAF-16(FOXO) are perhaps required for longevity (39). The modulation of HLH-30 (TFEB) nuclear localization may be a promising strategy to improve somatic maintenance (32). Simultaneous mutations in the IIS and mTOR pathways that produce a synergistic effect was reported recently. Using genome-wide translational state analysis and genetic screening, Lan et al. identified ribosomal protein genes and *cyc-2.1*, which encodes one of the worm cytochrome orthologs, as negative regulators of longevity (80). *Cyc-2.1* knockdown significantly extended lifespan by activating the intestinal mitochondrial unfolded protein response (UPR^mt^), mitochondrial fission, and AMPK (80). The influence of mitophagy is to extend the lifespan, thus, the role of mTOR mediated mitophagy in longevity needs further study.

Recent studies show that mTOR influence on lifespan also relies on epigenetic cues. Histone modification can regulate lifespan by acting on mTOR signaling pathways. In *C. elegans*, COMPASS H3K4me3 methyltransferase (methylation of Lys^4^ of histone H3) deficiency promotes fat accumulation and extends lifespan by targeting RSKS-1 (S6K) in the mTOR complex (28). H3K4me3 methyltransferases have homologs in humans. Mutations in the trithorax group (TrxG) can reduce the H3K4me3 level and, in turn, extend lifespan. Moreover, this influence is heritable for three generations even if the TrxG function is restored in the F1 progeny (81).

## Dietary Restriction Extends Lifespan in *C. elegans*

Since the initial discovery in 1935 that animals feeding on less food lived substantially longer (82), dietary restriction (DR) has been shown to increase lifespan and delay the onset of multiple age-related pathologies in a wide variety of metazoans (83). DR extends lifespan not mediated by a single linear pathway but by multifactorial processes. There are two hypotheses postulated in *C. elegans:* (1) DR reduces insulin/IGF-I signaling, and (2) DR reduces the metabolic rate (84). In the model of *C. elegans*, Pandit elucidated the complexity of gene regulation following the initiation of DR in *EAT-2* and defined the central role of PHA-4(FOXA) in this process, justifying its position as a robust genetic regulator of DR-induced longevity (40) (Figure 1). Siler also found that PHA-4 played a key role in regulating DR-mediated longevity in adult *C. elegans*. PHA-4(FOXA) is required for lifespan extension *via* DR, but not the extension resulting from reduced IIS *via daf-2(insr)* mutants (41), indicating that PHA-4(FOXA) may be a part of a pathway distinct from IIS (42). Increasing lifespan by reducing TOR signaling requires PHA-4(FOXA) and is mediated by the *rsks-1* gene, encoding the homolog of the mammalian SK61. This indicates that FOXA is a necessary downstream component of a TOR-mediated increase in lifespan. However, the precise mechanism and intermediates that control this remain to be determined (43).

Since DR affects both IIS signaling and mTOR signaling, it is important to delineate the contribution of each to overall lifespan extension. In worms, a further reduction in TOR activity does not generate further lifespan extension under some DR regimens, nor protect from lifespan reduction by dietary enrichment, suggesting that mTOR may mediate an effect on lifespan under certain forms of DR (85). Lowering the methionine levels suppresses mTOR pathway activity and prolongs lifespan, suggesting that these types of diets can influence the aging process (86).

The nutrient-sensing pathway is regulated at the lysosomal membrane by several proteins, and the deficiency of which triggers widespread aging phenotypes. In response to environmental conditions, the lysosomal nutrient-sensing complex controls the autophagy process *via* several factors, including the transcription factors TFEB and FOXO, which have previously been shown to be associated with lifespan extension (Figure 1). A major regulator of autophagy and lysosomal gene expression is HLH-30 (TFEB). HLH-30 (TFEB) is required for innate immunity and lifespan extension in different long-lived nematode mutants *via* the autophagic response to starvation. The nuclear localization of HLH-30 (TFEB) is modulated *via* phosphorylation by mTOR (44). This key metabolic pathway strongly depends on nucleocytoplasmic compartmentalization, a cellular phenomenon that is gradually lost with aging.

AAK-2 (AMPK) is another crucial metabolic energy sensor that links nutrient availability to lifespan (18). AMPK regulates mTORC1 activity and shares downstream effectors of lifespan modulation with mTOR (87). AMPK can also regulate mammalian FOXO3 (88). In worms, the activation of AMPK and its downstream metabolic targets often relies on the level of DR and the composition of the restricted diets (68). The response of AMPK to glucose and oxidants is glycogen-dependent (89). Moreover, AMPK signaling may provide a link between glucose toxicity, glycogen accumulation, oxidative stress, and aging. When activated by a drop in energy status, AMPK binds to AMP or ADP to promote ATP production. AMPK can bind and phosphorylate PGC-1α, FOXO, and SIRT1 (33). Especially, sirtuins as a specific group of histone NAD dependent deacetylases are associated with longevity (32). Deletion or inhibition of sirtuin SIR-2.1 (*C. elegans* ortholog of human SIRT1) reduces lifespan. DR could stimulate SIR-2.1 (SIRT1) and upregulates autophagy in *C. elegans* and human cells to extend lifespan (45). Moreover, human SIRT1, together with AMPK, could induce autophagy by upregulating autophagic genes and inhibiting mTOR signaling (46). These outcomes indicated that epigenetic regulation of lifespan was closely linked to cell metabolism and nutritional status in *C. elegans*.

## Drug Screening For Compounds That Extend Lifespan in Nematodes

The *C. elegans* model provides several advantages when performing chemical screening for the identification of drug candidates. This is especially true for primary drug screening, which involves relatively smaller spaces, lower costs, and time-consuming assessments. Nematodes can be inexpensively cultured in large quantities, and the relatively short lifespan of *C. elegans* ensures this organism provides high-throughput screening for anti-aging drug. Also, the effects of drugs can be tested directly in the whole organism, such that compounds that are toxic for development can be eliminated immediately. *C. elegans* can also be used for genetic analysis and investigations of chemical interventions for longevity. Moreover, a variety of assays suitable for high-throughput screening for anti-aging compounds are currently being developed (6, 90). Based on mutations in the *age-1(PI3K)* or *daf-2(INSR)* genes, and reduction in the *daf-16(foxo)* mutant, several compounds have been identified that significantly increase the lifespan of this nematode. Kumar demonstrated that *C. elegans* treated with 25 and 50 μM silymarin increased the mean lifespan of this organism by 10.1 and 24.8%, respectively, as compared to untreated controls (91). Another study demonstrated that fullerenol attenuated the endogenous levels of ROS and provided protection to *C. elegans* by up-regulating stress-related genes under stress conditions, which was in a DAF-16-dependent manner, thus improving lifespan (92).

Many potential chemical candidates for extending lifespan are currently being investigated, including the following aging modulating compounds: (1) metformin (biguanide anti-glycemic agent for AMPK activation), (2) rapamycin (immunosuppressing agent and mTOR inhibitor), (3) resveratrol (polyphenol and sirtuin activator), (4) spermidine (polyamine and inductor of autophagy), (5) aspirin (COX inhibitor, antithrombosis, and antioxidant), and (6) masoprocol (catechol with antioxidant and anti-inflammatory properties) (93). Active AMPK downregulates mTORC1 activity indirectly by phosphorylating the serine sites on TSC2, and directly by phosphorylating Raptor. The AMPK activating drug metformin (commonly prescribed to diabetic patients) was shown to increase lifespan in *C. elegans* (47). Similarly, metformin has been shown to act on mTOR signaling *via* Redd1, also independently of AMPK (94).

The toxicity ranking screening of *C. elegans* has been repeatedly found to be as predictive of rat LD_50_ ranking as mouse LD_50_ ranking. Additionally, many instances of the conservation of the mode of toxic action have been reported between mammals. These consistent correlations make a case for the inclusion of C. elegans assays in early safety testing and as one component in tiered or integrated toxicity testing strategies (95). These findings indicate that *C. elegans* could be a bridge between *in vitro* assays and mammalian toxicity testing by combining established *in vitro* handling techniques and cost ratios with oral toxicity test data from an intact organism. Given that nematodes lack most mammalian organs, it is unrealistic to expect that any combination of *C. elegans* assays alone will replace in-depth descriptive toxicology analyses in mammals. However, although organismal toxicity endpoints often differ, many pathways of toxicity and modes of toxic action are conserved between worms and humans (95).

Besides acting as markers for the genetic regulation during aging, epigenetic mechanisms may also be targets for drug screening in aging or age-related diseases (28). Researchers have confirmed this promising application. For example, resveratrol, as an activator of SIR2.1 (SIRT1) and AMPK, extends the lifespan of *C. elegans* (48). The *sir2* mutation could obliterate the effect of resveratrol. Natural compounds, such as curcumin or alkylresorcinols, enhance SIRT1 activity and have been confirmed to extend the lifespan (49). These findings indicated that SIR2.1 (SIRT1) could be a promising target for aging interventions. Overall, epigenetic research will be a powerful way for aging interventions of drugs. Lifespan extension for humans could be achieved by powerful genetic tools and further understanding of aging mechanisms in simple invertebrate models.

## Conclusion and Future Challenges

As a model system, the nematode *C. elegans* could be used for studying genetic approaches to understand the aging process, age-related diseases, mechanisms of longevity, and drug screening for compounds that increase lifespan. Longevity studies on this lower organism have helped provide an outline of the signaling pathways involved in aging and predicting their behavior in complex organisms. However, which molecular pathways are causative and which accompany aging need further research. Also, the mechanisms of epigenetic regulation associated with aging are still on the way to be elucidated in depth. In the future, disease models including nematodes and *C. elegans* will definitely provide further insights into the aging process.

## Author Contributions

ZL and GW: conceptualization and resources. SZ and TZ: methodology, literature arrangement, and writing - original draft preparation. SZ, ZL, and FL: writing - review and editing. ZL: funding acquisition. ZL, GW, and FL: supervision. All authors contributed to the article and approved the submitted version.

## Conflict of Interest

The authors declare that the research was conducted in the absence of any commercial or financial relationships that could be construed as a potential conflict of interest.

## Footnotes

^1^*What is C. elegans and Why Work on it? An Introduction for Those Unfamiliar With the Worm*. Available online at: https://cbs.umn.edu/cgc/what-c-elegans.
